# Human blood microRNA hsa-miR-21-5p induces vitellogenin in the mosquito *Aedes aegypti*

**DOI:** 10.1038/s42003-021-02385-7

**Published:** 2021-07-09

**Authors:** Hugo D. Perdomo, Mazhar Hussain, Rhys Parry, Kayvan Etebari, Lauren M. Hedges, Guangmei Zhang, Benjamin L. Schulz, Sassan Asgari

**Affiliations:** 1 Australian Infectious Disease Research Centre, School of Biological Sciences, The University of Queensland, Brisbane, QLD Australia; 2 School of Chemistry and Molecular Biosciences, The University of Queensland, Brisbane, QLD Australia

**Keywords:** miRNAs, Entomology

## Abstract

Mosquito vectors transmit various diseases through blood feeding, required for their egg development. Hence, blood feeding is a major physiological event in their life cycle, during which hundreds of genes are tightly regulated. Blood is a rich source of proteins for mosquitoes, but also contains many other molecules including microRNAs (miRNAs). Here, we found that human blood miRNAs are transported abundantly into the fat body tissue of *Aedes aegypti*, a key metabolic center in post-blood feeding reproductive events, where they target and regulate mosquito genes. Using an artificial diet spiked with the mimic of an abundant and stable human blood miRNA, hsa-miR-21-5p, and proteomics analysis, we found over 40 proteins showing differential expression in female *Ae. aegypti* mosquitoes after feeding. Of interest, we found that the miRNA positively regulates the *vitellogenin* gene, coding for a yolk protein produced in the mosquito fat body and then transported to the ovaries as a protein source for egg production. Inhibition of hsa-miR-21-5p followed by human blood feeding led to a statistically insignificant reduction in progeny production. The results provide another example of the involvement of small regulatory molecules in the interaction of taxonomically vastly different taxa.

## Abstract (web summary)

Perdomo et al. fed mosquitos (*Aedes aegypti*) a diet spiked with human blood miRNA, hsa-miR-21-5p and used proteomics analysis to show differential protein expression in female *Ae. aegypti* mosquitoes after feeding. They showed that hsa-miR-21-5p positively regulated vitellogenin, which plays a role in egg production, thus could have effects on *Ae. aegypti* progeny production.

## Introduction

The World Health Organization (WHO) reports that 1 million people die and over 1 billion get infected with vector-borne diseases every year^1^. Although vector-borne diseases are caused by different types of pathogens, and transmitted by various vectors with different modes of transmission, there is one commonality among them: the vectors of these diseases at one point of their life need to feed on vertebrate blood in order to transmit pathogens and reproduce.

Blood feeding is a trait that has evolved in various groups of arthropods throughout the evolutionary history^2^. Blood is a mixture of white blood cells, red blood cells and plasma. The red blood cells compose 45% of the blood volume and are the principal source of proteins^3^. The plasma accounts for the other 55% and is a good source of other nutrients such as salts, sugar, and lipids^4^. In mosquitoes, following the acquisition of a blood meal, several tightly regulated molecular processes culminate in oviposition^5–7^. Signals such as free amino acids circulating in the hemolymph, insulin-like peptides, juvenile hormone, and ecdysone are received by the fat body (analogous to the vertebrate liver), and this informs the tissue to start producing yolk protein precursors. These precursors are transported into the ovaries and serve as the exclusive protein source for egg production (reviewed in ref. ^8^). While potential effects of vertebrate host blood components, such as hemoglobin, pathogen-derived factors, complement, insulin and insulin-like growth factor-1, and TGF-b1 on mosquito physiology and immunity have been explored (reviewed in ref. ^9^), the likely effects of human microRNAs (miRNAs) present in the blood on mosquito biology have not been investigated.

miRNAs are a group of small noncoding RNAs ~22nt long that regulate gene expression at the post-transcriptional level^10,11^. They are present in vertebrates and arthropods in the cytosol, but can also exist as extracellular RNA (exRNA). Stable circulating miRNAs occur in human body fluids, including blood, in high concentrations, some of which are used as biomarkers in the diagnosis of various diseases and disorders^12–14^ or have been considered as potential therapies in clinical studies^15,16^. In human, 93 unique miRNA species were found in blood^17^. miRNAs are commonly packaged in membrane vesicles and macromolecular complexes^13,17^. Accumulating evidence suggests blood-circulating miRNAs (endogenous or exogenous) can affect other tissues or microorganisms exposed to them (reviewed in refs. ^18,19^). As there is not much variation among the most abundant miRNAs in the circulating blood, one can assume that every time a mosquito feeds on blood, it ingests approximately the same profile of human miRNAs (hsa-miRNAs)^20^.

*Aedes aegypti* is one of the most notorious mosquito species responsible for the transmission of several medically important viruses, such as dengue, Zika, Yellow fever, and chikungunya, and is expanding its geographical distribution^21^. The mosquito has become completely urbanized, feeding almost exclusively on humans. *Ae. aegypti* is an anautogenous mosquito, which means it acquires all the nutrients needed to produce its eggs from a blood meal^22^. In this study, we investigated if hsa-miRNAs present in the human blood are transported through the midgut of the mosquito into the fat body and if they have any effect on the mosquito’s gene expression profile and proteome structure, particularly in view of reproduction, which is a key outcome of blood feeding.

## Results

### Bioinformatic detection of vertebrate miRNAs in mosquitoes

**Fig. 1:**
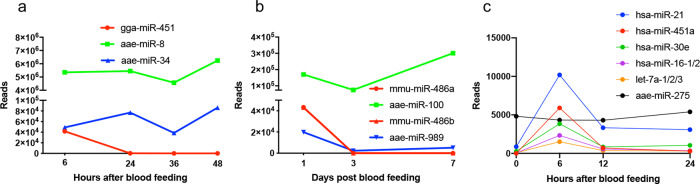
Vertebrate miRNAs are abundant in *Ae. aegypti* after blood feeding. Examples of reads of different miRNAs in deep sequencing data from **a** the fat body of *Ae. aegypti* mosquitoes fed on chicken blood^23^, and **b** *Ae. aegypti* whole body fed on mouse blood^24^. miRNAs with prefix aae, mmu, and gga represent miRNAs from *Ae. aegypti*, mouse, and chicken, respectively. **c** Analysis of small RNA-Seq data revealed the presence of the top five most abundant human miRNAs in the dissected fat body of mosquitoes after blood feeding with the peak of abundance at 6 h after blood feeding. Each dot point represents the average of three biological replicates. aae-miR-275 is included as an endogenous *Ae. aegypti* miRNA for comparison.

Considering the presence of vertebrate miRNAs in blood, we wanted to find out if these miRNAs can be detected in mosquitoes fed on blood, and whether they pass through the mosquito midgut to be subsequently translocated into other mosquito tissues. For this, we first took advantage of the RNA-Seq data available in public domains. In a study on *Ae. aegypti* that explored the effect of blood feeding on the miRNA profile of the mosquito, the fat body tissue was dissected at different hours post-feeding and subjected to small RNA-Seq analysis^23^. The mosquitoes were fed on chicken blood. We analyzed these small RNA libraries and found abundant numbers of chicken-specific miRNAs in the fat body of the mosquitoes, in particular gga-miR-451, as early as 6 h after blood feeding (about 40,000 reads comparable to the mosquito aae-miR-34, second highly abundant miRNA) which declined over time (Fig. 1a). The result shows that (1) blood-specific miRNAs pass through the midgut and are translocated into the fat body tissue, and (2) the miRNAs are found in large numbers that could be biologically relevant. Analysis of RNA-Seq data from another study^24^, in which *Ae. aegypti* mosquitoes were fed on mouse blood, showed that mosquitoes contained large numbers of reads corresponding to mouse-specific miRNAs, in particular, mmu-miR-486a and mmu-miR-486b, one day after blood feeding, which similarly declined over time (Fig. 1b). As shown in Fig. 1a and b, the abundances of the blood-derived miRNAs are comparable to endogenous *Ae. aegypti* miRNAs. None of those vertebrate miRNAs that were found in blood-fed mosquitoes (from both studies) were found in non-blood-fed mosquitoes, indicating that the detected miRNA reads that we found in blood-fed mosquitoes were genuine and not artifacts. Of note, it appears that the abundances of chicken blood miRNAs in *Ae. aegypti* fat body (Fig. 1a) were higher than those in the fat body of mosquitoes fed on human blood (Fig. 1c). However, this could be due to artifacts related to preparation of RNA-Seq libraries in the two studies, and the depth of sequencing, which require further investigation.

### Detection of hsa-miRs in *Ae. aegypti* fat body

In order to find out if human miRNAs are translocated into the *Ae. aegypti* fat body tissue after blood feeding, mosquitoes were fed on a volunteer. The fat body of the mosquitoes was dissected from those collected just before blood feeding (NBF; negative control) and 6, 12, and 24 h post blood feeding (hpb). Small RNAs were extracted from the tissue samples and subjected to small RNA-Seq. To analyze the data, the raw reads were first trimmed and cleaned and subsequently mapped to the *Ae. aegypti* genome AaegL5.2. The unmapped reads were used to detect the presence of hsa-miRNAs based on reported human miRNAs on miRBase. Figure 1c shows the presence of the five most abundant human blood miRNAs in the mosquito fat body tissue. Noticeably, all of them had a peak of abundance at 6 hpb, which substantially declined by 12 hpb, although hsa-miR-21-5p remained present until 24 hpb. BLASTn analysis confirmed that these mature miRNAs were absent from the *Ae. aegypti* genome. The read numbers of aae-miR-275, which has been shown to be an abundant vitellogenic *Ae. aegypti* miRNA in the fat body^25^, is shown for comparison.

**Fig. 2:**
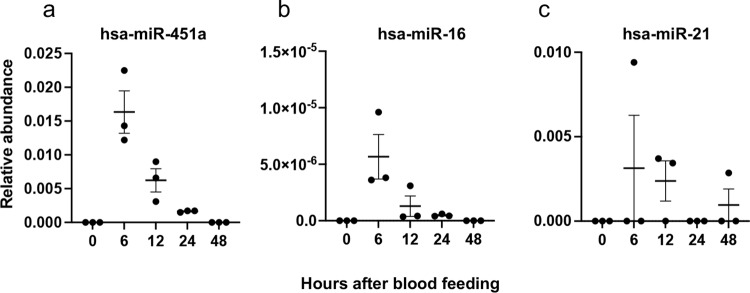
Presence of human miRNAs in *Ae. aegypti* fat body as determined by RT-qPCR. The relative abundance of **a** hsa-miR-451a, **b** hsa-miR-16-5p, and **c** hsa-miR-21-5p in the fat body of *Ae. aegypti* after blood feeding using *Ae. aegypti* U6 non-coding small nuclear RNA as reference. Each point represents the mean of three biological replicates; each replicate is a pool of fat bodies from seven different mosquitoes. Error bars represent standard error of the mean (SEM) in three biological replicates.

As a complementary approach to sRNA-Seq, RT-qPCR detection in mosquito fat body was carried out for different human miRNAs that are known to be highly abundant in human blood^17^. Of those, we could successfully amplify hsa-miR-451a, hsa-miR-16-5p, and hsa-miR-21-5p (Fig. 2), which showed a peak at 6 hpb and by 48 h had declined to under the detection limit.

### Differential expression of *Ae. aegypti* genes after hsa-miR-21-5p mimic feeding

**Fig. 3:**
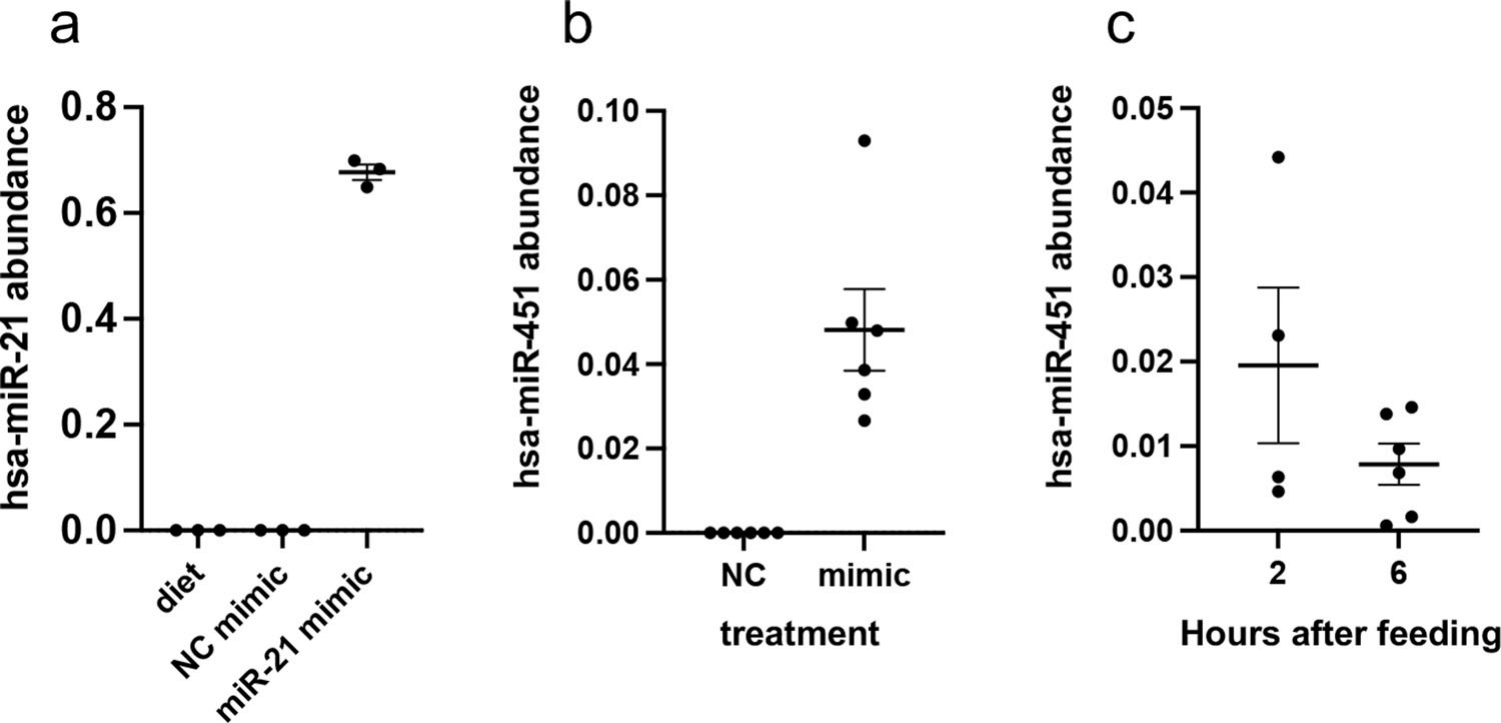
Human miRNA mimics are transported into the mosquito fat body. RT-qPCR detection of hsa-miR-21-5p **a** 12 h after feeding, **b** hsa-miR-451a 12 h after feeding and **c** 2 and 6 h after feeding, on artificial diet spiked with its mimic. NC, negative control mimic. Two separate experiments were performed; each point represents the fat body of one mosquito. *Ae. aegypti* U6 non-coding small nuclear RNA was used as reference. Error bars represent standard error of the mean (SEM) in three or more biological replicates.

In order to determine if the expression of mosquito genes could be regulated due to the ingestion of human miRNAs, we focused mainly on hsa-miR-21-5p considering its high abundance, transfer to the fat body, and longer persistence in mosquitoes compared to other human blood miRNAs (Fig. 1c). As blood contains a mixture of various human miRNAs, to specifically find the effect of hsa-miR-21-5p on mosquito gene expression, 4-day-old female mosquitoes were fed on an artificial diet that consisted of proteins in a physiological solution spiked with miRNA mimics or a negative control mimic. The first step was to find out if the mimic was transported into the fat body. Twelve hours after feeding, fat body was dissected from mosquitoes, and the mimic abundance was quantified. We could detect hsa-miR-21-5p only in the group that had ingested artificial diet spiked with its mimic and not in the control group fed with the negative control mimic (Fig. 3a). For mimic transfer, we also tested mosquitoes after feeding another human miRNA mimic, hsa-miR-451a. We could detect the mimic of hsa-miR-451a in the fat body at 12 h after feeding (Fig. 3b), but also at 6 h and as early as 2 h after feeding in the fat body (Fig. 3c). Notably, the relative amount of hsa-miR-451a in the fat body of mosquitoes fed on the artificial diet spiked with the mimic at 6 h post-feeding (Fig. 3c) was comparable to that in the fat body of mosquitoes fed on human blood (Fig. 2a).

**Fig. 4:**
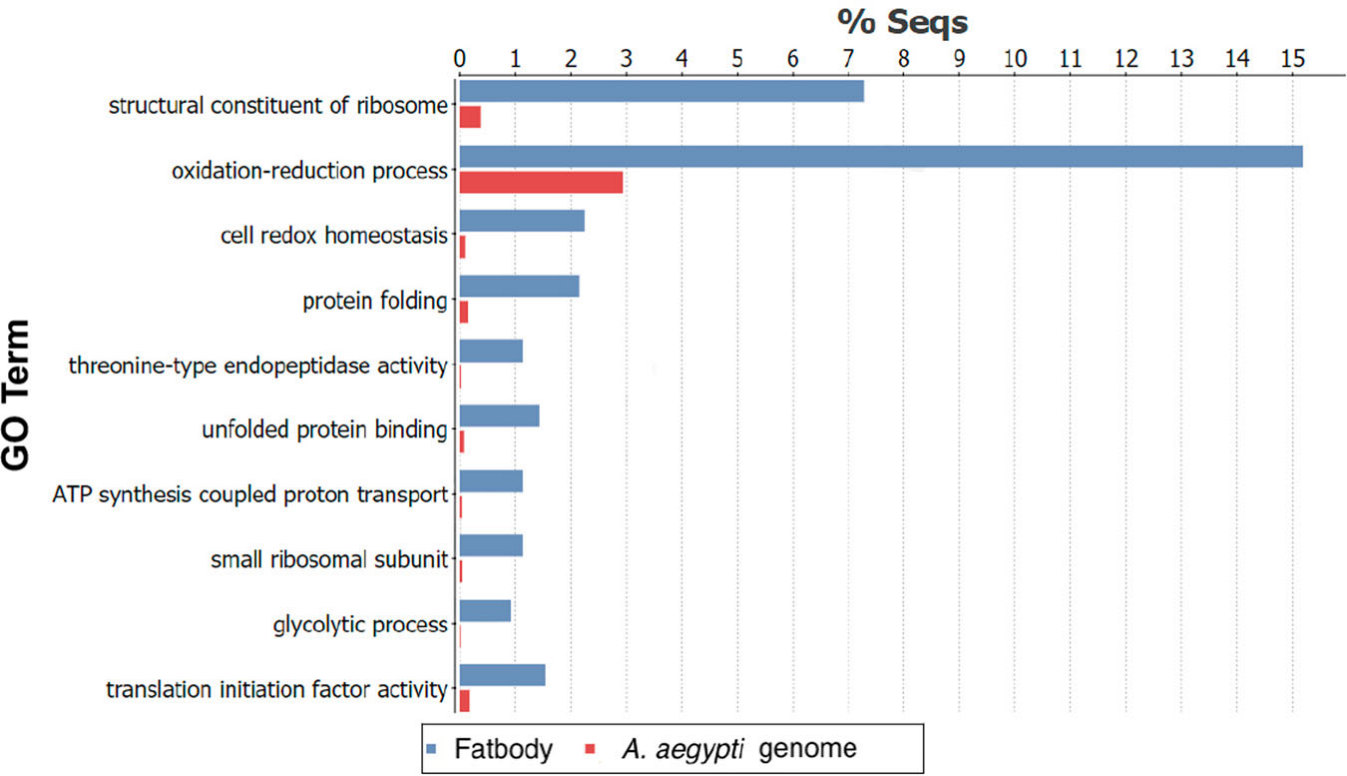
Gene ontology analysis of *Ae. aegypti* fat body proteins. Bar chart showing over-represented GO terms present among detectable proteins in *Ae. aegypti* fat body 12 h after feeding on artificial diet.

Considering successful transport of hsa-miR mimics into the mosquito’s fat body and that miRNAs are known to regulate gene expression mainly at the translational level, in the next step, we carried out a proteome analysis of the fat body. To investigate how the proteome of the fat body of *Ae. aegypti* is modulated by hsa-miR-21-5p, our first step was to determine what proteins are produced in the fat body after feeding on the artificial diet. Proteins were extracted from the fat body of female mosquitoes 12 h after feeding, processed, and identified by LC-MS/MS, using ProteinPilot to identify proteins. Overall, 995 proteins were detected with a 5% global false discovery rate (FDR) (Supplementary Data 1). Using the software OmicsBox, a gene ontology analysis of these proteins was carried out with structural components of the ribosome, oxidation-reduction process, and cell redox homeostasis, the three terms that were enriched at higher percentages (Fig. 4).

**Fig. 5:**
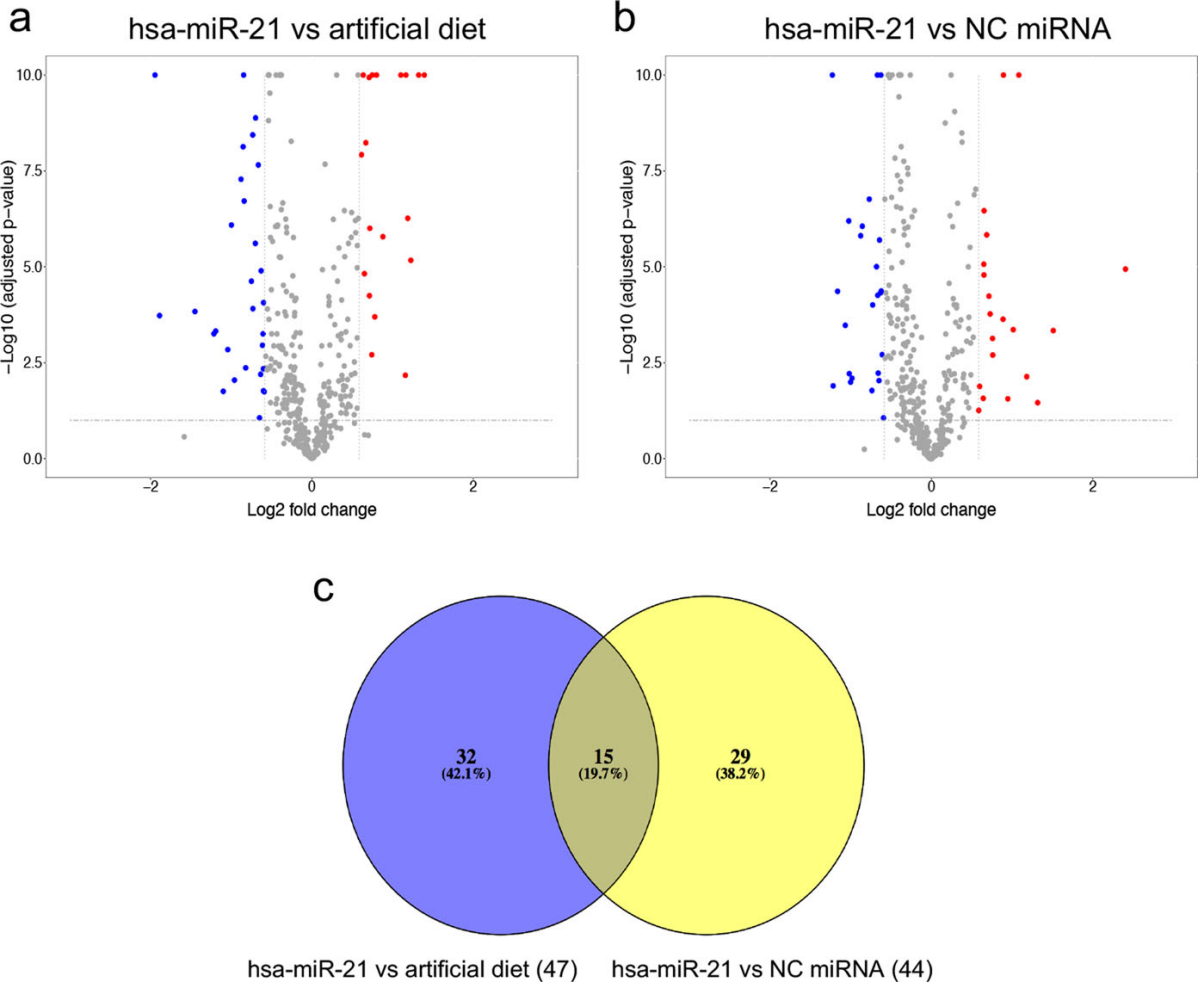
hsa-miR-21-5p mimic modulates the proteome of the fat body in *Ae. aegypti*. *Ae. aegypti* mosquitoes were fed on artificial diet only, an artificial diet with NC mimic, or artificial diet with hsa-miR-21 mimic. Fat body was dissected 12 h after feeding; proteins were extracted, processed and identified using LC-MS/MS. Quantifiable proteins were used to determine differentially abundant proteins between **a** hsa-miR-21 mimic and artificial diet fed mosquitoes, and **b** hsa-miR-21 and NC mimic fed mosquitoes. Differentially abundant proteins were considered when they had an adjusted *p* value <0.05 and a fold change >1.5. **c** A Venn diagram of differentially abundant proteins from (**a**, **b**) and the overlaps. Three biological replicates per treatment were used for the proteomics analysis.

SWATH-MS was then used to measure the relative abundance of each protein. Using PeakView, 566 proteins were quantified with a 1% FDR cutoff and an Extracted Ion Chromatogram (XIC) extraction window of 15 min. This means that of the 995 proteins that were detectable in the tissue, only 566 were quantifiable. Using the intensity of the quantifiable proteins, MSstats was used to determine differentially abundant proteins. We looked for proteins that had a differential abundance in hsa-miR-21-5p mimic fed mosquitoes against mosquitoes fed with artificial diet, and hsa-miR-21 mimic fed mosquitoes against mosquitoes fed with a negative control mimic (NC). Differential abundance was considered significant when a protein had an adjusted *p* value lower than 0.05 and an absolute fold change higher than 1.5. We then compared the proteins that demonstrated differential abundance and analyzed only the ones that were present in both comparisons (Fig. 5). Overall, 47 proteins were found to be differentially abundant when hsa-miR-21 treatment was compared against artificial diet only, and 44 when hsa-miR-21 was compared against NC miRNA mimic. Of those, only 15 were present in both comparisons that are listed in Table 1. Eight proteins were less abundant and seven more abundant. We performed a GO term overrepresentation analysis of the 15 proteins, but no enriched term was identified. Of special interest was the presence of vitellogenin among the upregulated proteins.

**Table 1:** Differentially abundant proteins in the fat body of *Ae. aegypti* after feeding on artificial diet supplemented with hsa-miR-21-5p mimic.

	hsa-miR-21 vs diet		hsa-miR-21 vs NC mimic		
Protein ID	Log_2_ FC	adj. *p* value	Log_2_ FC	adj. *p* value	Gene description
AAEL025608-PA	−1.94	0.00	−1.23	0.00	Putative cuticle protein
AAEL025680-PA	−1.10	1.76E−02	−1.22	1.27E−02	*Aedes aegypti* enolase-phosphatase E1
AAEL006836-PB	−1.00	8.14E−07	−1.02	6.36E−07	Dihydropteridine reductase
AAEL003750-PA	−0.96	8.99E−03	−1.02	6.10E−03	Predicted: *Aedes aegypti* nucleoplasmin-like protein
AAEL004151-PF	−0.85	7.39E−09	−0.64	2.00E−06	60S ribosomal protein L29
AAEL003193-PB	−0.63	1.27E−05	−0.77	1.72E−07	Inorganic pyrophosphatase
AAEL019604-PC	−0.61	1.11E−03	0.60	1.31E−02	*Aedes aegypti* synaptic vesicle membrane protein VAT-1 homolog-like
AAEL005593-PC	−0.59	1.81E−02	−0.61	1.95E−03	Predicted: *Aedes aegypti* uncharacterized
AAEL012326-PA	0.65	1.51E−05	0.65	1.63E−05	Calmodulin
AAEL003130-PD	0.71	5.66E−05	0.71	5.81E−05	Bcr-associated protein, bap
AAEL010127-PA	1.10	0.00	1.08	0.00	Pupal cuticle protein 78E, putative
AAEL025845-PA	1.16	6.72E−03	0.76	1.99E−03	Predicted: *Aedes aegypti* uncharacterized
AAEL001390-PA	1.16	3.90E−14	0.65	3.45E−07	Predicted: *Aedes aegypti* bifunctional endo-1,4-beta-xylanase XylA
AAEL006126-PA	1.23	6.76E−06	0.76	7.36E−04	Predicted: *Aedes aegypti* vitellogenin-A1-like
AAEL017563-PA	1.32	0.00	0.89	7.42E−13	Predicted: *Aedes aegypti* bifunctional endo-1,4-beta-xylanase XylA

### hsa-miR-21-5p upregulates *Ae. aegypti* vitellogenin

**Fig. 6:**
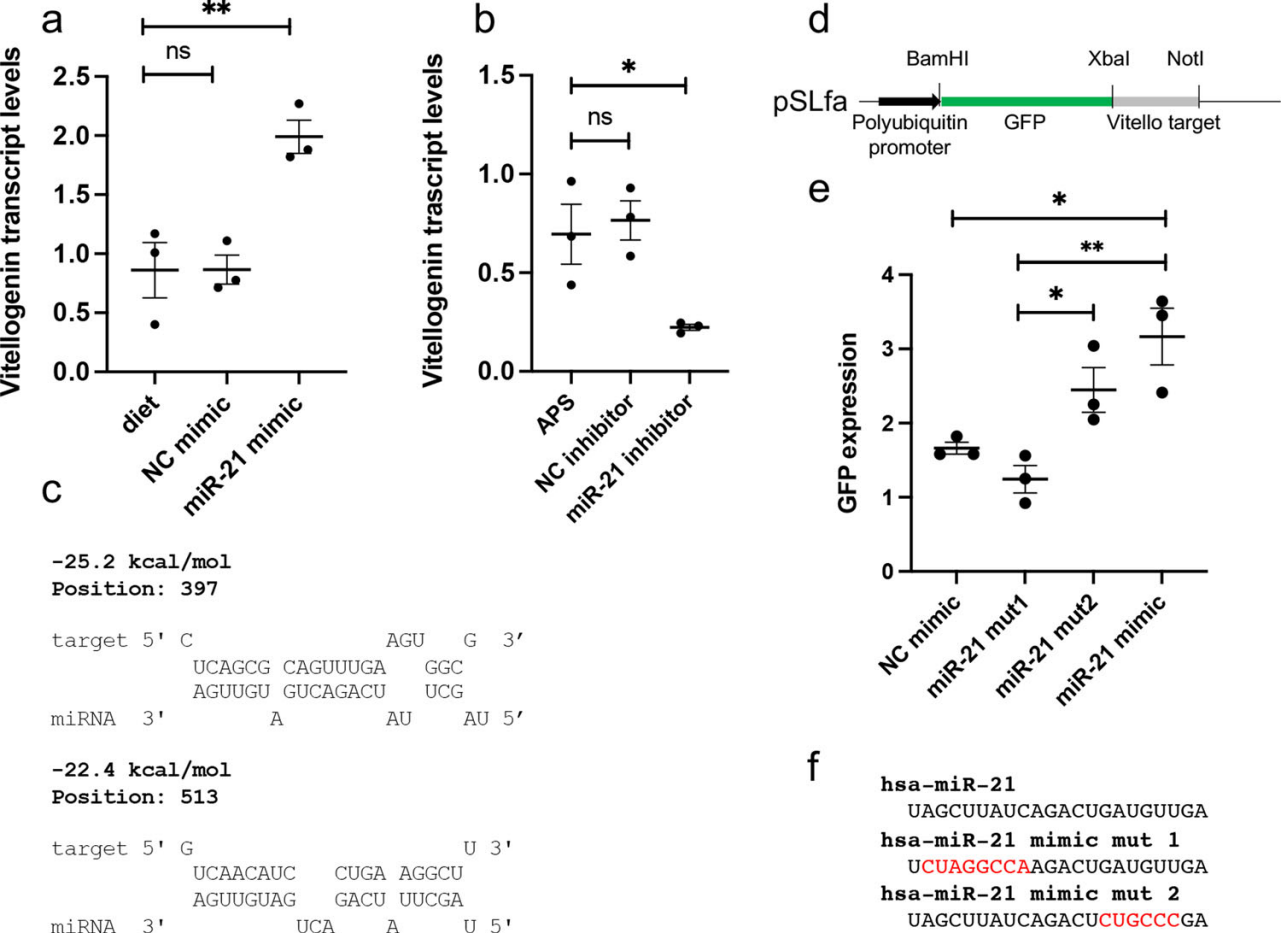
Vitellogenin is a direct target and is upregulated by hsa-miR-21-5p. **a** Mosquitoes were fed with artificial diet only or on diet containing NC mimic or hsa-miR-21-5p mimic. They were collected 12 h after feeding, and *vitellogenin* levels relative to *RPS17* were determined using RT-qPCR. Each data point represents a single mosquito. One-way ANOVA with Tukey’s post-hoc multiple comparisons were used for data analysis. ns, not significant; **p < 0.01. **b** Mosquitoes were injected with APS buffer, a negative control (NC) inhibitor or hsa-miR-21-5p inhibitor. Four days after injection mosquitoes were fed on a human volunteer and 12 h after feeding levels of *vitellogenin* relative to *RPS17* were detected using RT-qPCR. One-way ANOVA with Tukey’s post-hoc multiple comparisons were used for data analysis. ns, not significant; *p < 0.05. **c** Bioinformatic analysis using RNAHybrid (v2.2.1) showing two different target sites in the *vitellogenin* gene. **d** The *vitellogenin* target sequences were cloned downstream of *GFP* in the pSLfa vector. **e** Aag2 cells were co-transfected with the plasmid and NC mimic, hsa-miR-21-5p mimic, or two different mutant mimics. *GFP* transcript levels relative to *RPS17* were determined by RT-qPCR 48 h after transfection. One-way ANOVA with Tukey’s post-hoc multiple comparisons were used for data analysis. *p < 0.05; **p < 0.01. **f** The sequences of hsa-miR-21-5p and mutated residues (red residues) are shown below the graph. Error bars in all the graphs represent standard error of the mean (SEM) in three biological replicates.

Vitellogenin is the major yolk protein in insects, which is highly induced and synthesized in the fat body of mosquitoes following a blood meal^26^. Considering the increased abundance of vitellogenin protein in the fat body of mosquitoes after feeding on hsa-miR-21-5p mimic based on proteomics analysis, we also examined the transcript levels of the *vitellogenin* gene in *Ae. aegypti* mosquitoes following feeding on artificial diet spiked with hsa-miR-21-5p mimic. RT-qPCR analysis of the fat body tissue 12 h after mimic feeding revealed significant (One-way ANOVA *p* = 0.0054) up-regulation of *vitellogenin* transcripts in mosquitoes fed with hsa-miR-21-5p mimic compared to the NC mimic with scrambled sequence and diet only (Fig. 6a). The result suggested a positive correlation between hsa-miR-21-5p and *vitellogenin*. To further confirm this correlation, female mosquitoes 3–4 days after emergence were injected with either an inhibitor of hsa-miR-21-5p, NC scrambled sequence inhibitor or *Aedes* physiological solution (APS). The reason for injection of the inhibitor rather than feeding was that our attempts to inhibit miRNAs through feeding miRNA inhibitors resulted in no miRNA inhibition compared to injection. Further, we wanted the blood feeding to be as natural as possible without addition of an exogenous compound. Injection of the inhibitor also ensures that the inhibitor is already present in the mosquito prior to blood feeding and arrival of hsa-miR-21-5p. Four days after injection, the mosquitoes were fed on human blood and collected 12 h after feeding. RT-qPCR analysis revealed significantly (One-way ANOVA *p* = 0.0212) less induction of vitellogenin in hsa-miR-21-5p inhibitor-fed mosquitoes compared to those injected with APS or NC inhibitor (Fig. 6b).

To find out if hsa-miR-21-5p could directly target *Ae. aegypti vitellogenin* mRNA, we searched for potential target sites of the miRNA in the *vitellogenin* transcript with RNAHybrid (v2.2.1) with the default parameters^27^. Two potential target sites were identified in the open reading frame of the *vitellogenin* gene (Fig. 6c). The *vitellogenin* target sequences covering both sites and spanning 300 nucleotides were cloned downstream of a *GFP* reporter gene in the pSLfa vector (Fig. 6d). The plasmid was co-transfected into Aag2 cells together with hsa-miR-21-5p or a control miRNA. Compared to the NC mimic with scrambled sequences, *GFP* expression was significantly (One-way ANOVA *p* = 0.0038) induced with hsa-miR-21-5p (Fig. 6e). To ensure sequence specificity, two mutant hsa-miR-21-5p mimics were generated and included in the experiment, mutant 1 with mutated seed region and mutant 2 with mutations towards the 3’ end of the miRNA (Fig. 6f). While induction of *GFP* was abolished in mutant 1 mimic transfected cells, mutant 2 was able to induce *GFP* expression, but to a lesser extent than the wild-type mimic (Fig. 6e). The results suggested that (1) hsa-miR-21-5p directly interacts with the *vitellogenin* transcripts, and (2) the miRNA positively regulates the target consistent with the results found in mosquitoes.

### Effect of inhibition of hsa-miR-21-5p on progeny production

**Fig. 7:**
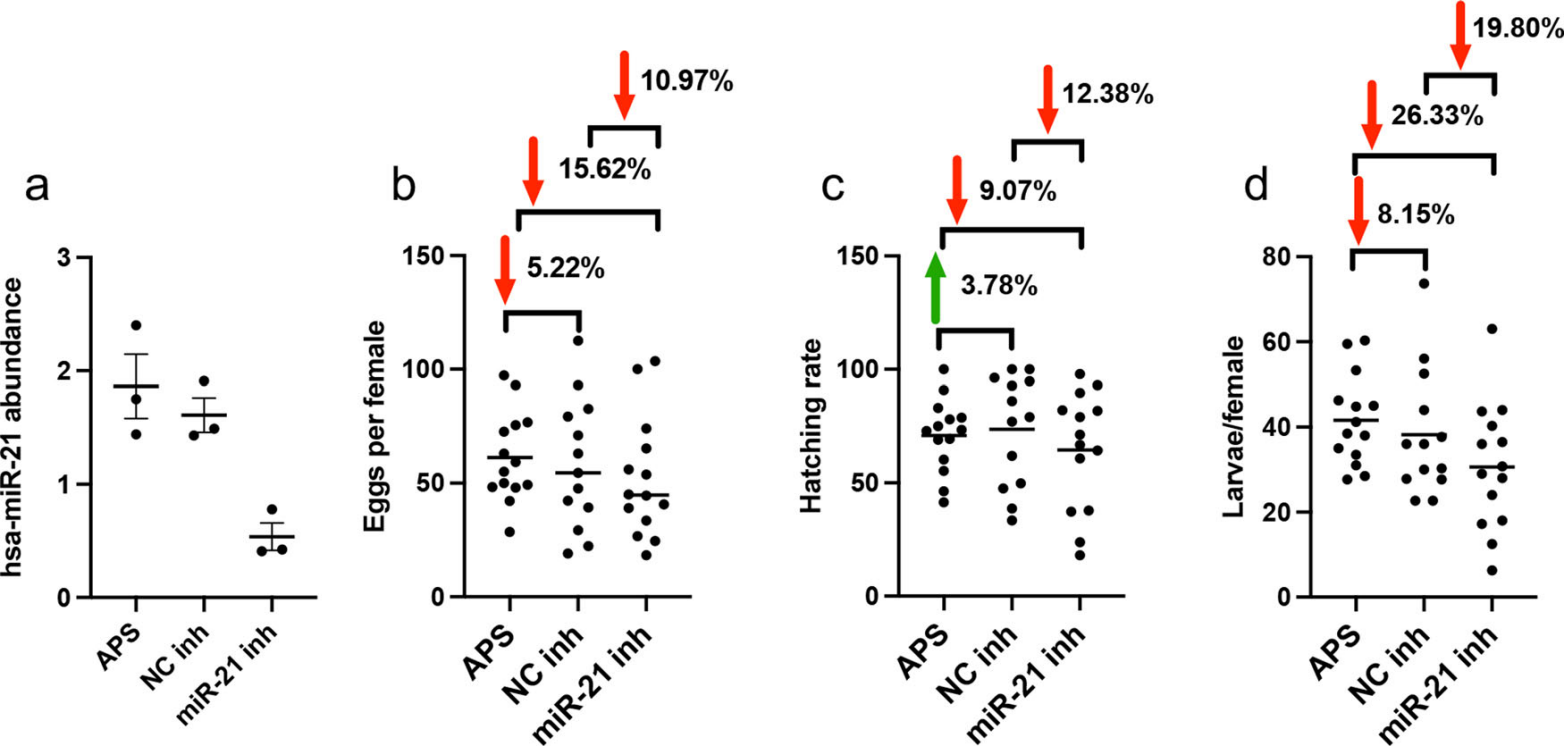
Inhibition of hsa-miR-21-5p leads to a reduction in the number of mosquito progeny. Four-day-old *Ae. aegypti* females were injected with hsa-miR-21-5p inhibitor, negative control inhibitor or APS buffer only. Four days after injection, mosquitoes were fed on a human volunteer. Between two to seven mosquitoes per replicate were pooled into a cage. Subsequently, the number of eggs deposited, eggs hatch rate, and the number of larval progenies were determined per cage and averaged per female. **a** A group of mosquitoes was collected 12 h after feeding to confirm inhibition of hsa-miR-21-5p. RNA was extracted from the mosquitoes, and the detection of hsa-miR-21-5p was carried out with RT-qPCR. *Ae. aegypti* U6 non-coding small nuclear RNA was used as reference. Error bars represent standard error of the mean (SEM) in three biological replicates. **b** The number of eggs deposited per female. Percent reduction (red arrow) in the number of deposited eggs is shown for each treatment. **c** Larva hatch rates are shown for each treatment. Percent reduction (red arrow) or increase (green arrow) in egg hatching is shown for each treatment. **d** The total number of larvae produced per female. Percent reduction (red arrow) in the number of larvae per female is shown for each treatment. The experiment was repeated five times independently with similar trends obtained. Each point represents the average of each pool. Upon testing, data in **b**–**d** showed normal distribution. One-way ANOVA with Tukey’s post-hoc multiple comparisons were used for data analysis.

Producing eggs and progeny is the principal reason for blood feeding in female mosquitoes, including *Ae. aegypti*. To investigate the role of hsa-miRNAs in this process, female mosquitoes were injected with hsa-miR-21-5p inhibitor, control inhibitor, and APS buffer only. Four days after injection, mosquitoes were fed on human blood and then egg deposition, hatch rate, and larval progeny were counted. RT-qPCR analysis of the extracted RNA from mosquitoes 12 h after injection confirmed suppression of hsa-miR-21-5p compared to those injected with the control inhibitor (−66%) or buffer only (−71%) (Fig. 7a). While there were no statistically significant reductions in the number of deposited eggs, hatch rate, and larval progeny between the treatments, interesting biological differences were noticed. There were on average 10.94% and 15.62% reductions in oviposition when NC inhibitor-injected mosquitoes were compared to those injected with hsa-miR-21-5p inhibitor, and APS-injected mosquitoes with those injected with hsa-miR-21-5p inhibitor, respectively (Fig. 7b). On the other hand, there was 5.22% decrease in oviposition when APS-injected mosquitoes were compared to those injected with NC inhibitor (Fig. 7b).

When egg hatch rate was considered, there were 13.36% and 12.07% reductions in egg hatching when NC inhibitor-injected mosquitoes were compared to those injected with hsa-miR-21-5p inhibitor, and APS-injected mosquitoes with those injected with hsa-miR-21-5p inhibitor, respectively, while only 3.87% increase in the hatch rate was observed when APS-injected mosquitoes were compared to those injected with NC inhibitor (Fig. 7c). Larval progeny production per female, which is affected by egg numbers and hatch rate showed a more profound compound response to injections. There were on average 19.80% and 26.36% reductions in the number of larvae per female when APS-injected mosquitoes were compared to those injected with hsa-miR-21-5p inhibitor, and NC inhibitor-injected mosquitoes with those injected with hsa-miR-21-5p inhibitor, respectively (Fig. 7d). In contrast, there was only 8.15% reduction in larval progeny when APS-injected mosquitoes were compared to NC inhibitor-injected mosquitoes. This suggests that hsa-miR-21-5p may contribute to egg production by upregulating the *vitellogenin* gene.

## Discussion

The study of RNA has shifted in the past two decades with discoveries that categorize RNA molecules not only as messengers but also as regulatory molecules in the cell. Furthermore, since the discovery that sRNAs are exported and transferred from cell to cell, their regulatory nature has been shown in cells residing in different tissues that are apart by long distances; for example from roots to shoots^28,29^. The regulatory nature of sRNAs is not restricted to one specific species, as it has been shown that cross-kingdom regulation can occur between different species. For example, hsa-miRNAs were found inside *Plasmodium falciparum* and shown to inhibit the parasite’s growth^30^. A recent study showed that the saliva of *Anopheles coluzzii* contains a subset of miRNAs that mimicked human miRNAs and could potentially target immune and inflammatory responses in humans^31^. *Ae. aegypti* is not foreign to cross-kingdom communication either. sRNAs from the endosymbiotic bacterium *Wolbachia* were found in *Ae. aegypti* and shown to regulate gene expression^32^. The entomopathogenic fungus *Beauveria bassiana* also introduces a miRNA-like molecule into *Ae. aegypti* and hijacks the RNAi machinery^33^. In this study, to the best of our knowledge, we show for the first time the uptake of hsa-miRNAs through blood feeding and their transport into the fat body of *Ae. aegypti* where they regulate mosquito gene expression.

Initially, based on publicly available data^23,24^, we identified vertebrate miRNAs in blood-fed *Ae. aegypti* mosquitoes, in particular in the fat body of the mosquito. Subsequently, we confirmed that human blood miRNAs can also pass through the mosquito midgut and reach the fat body tissue in abundant numbers. Their peak of abundance was at 6 h post blood meal which declined over time. Among blood miRNAs, hsa-miR-21 was found to be more persistent over time, and hence we focused our follow up studies on this miRNA. The effects of hsa-miR-21 has been studied in humans extensively, which is mostly associated with cancer. Dysregulation of the miRNA leads to cell proliferation and poor prognosis in cancers. hsa-miR-21 is upregulated in cancer cells, leading to down-regulation of target genes such as IGFBP3 and FBX011 (reviewed in refs. ^34^). This miRNA has also been considered as a biomarker for cancers^35,36^.

To further explore the effects hsa-miRNAs might have in *Ae. aegypti*, hsa-miR-21 mimics were added to a previously developed artificial diet, and the effects at the proteome-wide level were assessed at 24 h post feeding. Among the upregulated proteins, vitellogenin was identified, which is also highly induced at 24 h post blood feeding^37^. As part of yolk proteins induced following blood feeding and transported into the ovaries, vitellogenin is critical for oocyte formation^38^. Besides elevation at the protein level, *vitellogenin* transcript levels also significantly increased in hsa-miR-21 mimic-fed mosquitoes. Conversely, when mosquitoes were injected with hsa-miR-21 inhibitors and then fed on human blood, induction of *vitellogenin* was suppressed. The sequence-specific and direct interaction of hsa-miR-21 with target sequences in the vitellogenin mRNA were also confirmed using a reporter gene. Altogether, these results suggest positive regulation of vitellogenin by hsa-miR-21 both at the transcript and protein levels.

Positive regulation of genes by miRNAs is not unprecedented^39–43^. In *Ae. aegypti*, aae-miR-275 positively regulates *sarco/endoplasmic reticulum Ca2*+ *adenosine triphosphatase*^44^, and aae-miR-2940-5p positively regulates *metalloprotease m41 ftsh*^43^ and *arginine methyltransferase 3*^45^ by direct targeting. While the mechanism of positive regulation by miRNAs has not been explored in every example, those that have been investigated include induction of translation by binding to the 5′UTR region^46^, abolishing a stem-loop structure in mRNA that would otherwise hinder translation^47^, targeting transcripts of nonsense-mediated decay machinery^42^, and mRNA stabilization by rescuing it from exoribonuclease XRN1^48^.

Considering vitellogenin plays a significant role in egg production in mosquitoes^38^, and hsa-miR-21 induces it via blood feeding, we hypothesized that inhibition of the miRNA may affect the fecundity of *Ae. aegypti*. While we did not find statistically significant impacts of inhibition of the miRNA compared to the controls, we found a trend in reductions in progeny production. Overall, there was on average about 20–26% reduction in the number of larvae produced per female mosquito in which hsa-miR-21 was inhibited. It is worth mentioning that inhibition efficiency of hsa-miR-21 was around 70%. This suggests that while hsa-miR-21 is not the main factor in the induction of vitellogenin, it contributes to its induction, which could be an evolutionary consequence or adaptation of mosquitoes primarily feeding on human blood. It is known that miRNAs generally function as fine tuners that balance levels of gene expression^49^.

In summary, we showed that blood miRNAs are stable and transferred from midgut to the fat body of *Ae. aegypti* mosquitoes within a short period of time and in biologically relevant numbers. Concentrating on one of those miRNAs, hsa-miR-21, proteomics analysis showed differential abundance of several mosquito proteins in the fat body after feeding on miRNA-spiked artificial diet. Vitellogenin was found to be one of the targets of hsa-miR-21, which is positively regulated by the miRNA. Inhibition of the miRNA prior to blood feeding led to a trend in reductions in mosquito progeny production, although not statistically significant. While we only analyzed one of the human blood miRNAs, there are a number of other highly abundant blood miRNAs which could potentially target other mosquito genes. Further, we found a number of miRNAs that are identical or have the exact same seed region to the endogenous mosquito miRNAs, such as hsa-miR-92a-3p with 100% identity, that could affect the targets of the endogenous miRNA following an increase in their abundance after blood feeding. This study adds to the growing number of examples of small noncoding RNAs playing roles in cross-species interactions.

## Methods

### Cell lines and mosquito maintenance

*Ae. aegypti* Innisfail mosquitoes collected in the town of Innisfail, North Queensland, in April 2016 were maintained at 28 °C, 60% relative humidity and 12 h light/12 h dark cycling regime. Eggs were hatched in a plastic cup filled with water and 2 days later 300 larvae were counted and transferred to a tray with 3 L of water. Larvae were fed with Tropical Color Flakes (Tetra) ad libitum. After eclosion, mosquitoes were fed 10% sugar water ad libitum.

*Ae. aegypti* Aag2 cells were maintained in a mixture of 1:1 Schneider’s *Drosophila* Medium (Invitrogen) and Mitsuhashi and Maramorosch Insect Medium (Himedia), 10% fetal bovine serum and 1% penicillin/streptomycin.

### *Ae. aegypti* blood feeding, artificial diet feeding and injection

Blood feeding of mosquitoes was done directly from the calf of a volunteer (Human ethics approval number 2018000351). Parafilm was used as membrane and 37 °C water was circulated through the feeders to maintain a constant temperature. The composition of the artificial diet was as previously described^50^. In order to make the sorting of fed females easier, 0.002% (v/v) of blue food dye was added to the artificial diet. Briefly, 200 mg/mL bovine serum albumin (BSA) and 50 mM ATP were added to *Aedes* physiological solution (APS; 150 mM sodium chloride, 4 mM potassium chloride, 0.1 mM sodium bicarbonate, 0.6 mM magnesium chloride, 1.7 mM calcium chloride, 25 mM HEPES Buffer at a pH of 7.0). miRNA mimics or their controls (random sequence) were added at 100 μM to the diet.

For injection of miRNA inhibitors, 3–4-day-old female mosquitoes were chilled on ice for 5 min. Using a Nanojet III (Drummond) and pulled glass needles, 125  nL of 200 μM solution of hsa-miR-21-5p inhibitor or control inhibitor in APS were injected into anaesthetized mosquitoes. Four days after injection, mosquitoes were fed on human blood. After feeding, 2-7 mosquitoes were pooled in the same cage. The eggs were counted per cage and then averaged by the number of mosquitoes. Same was applied for hatching rate and larvae per female. miRNA mimics, inhibitors and controls were synthesized by Genepharma.

### RNA extraction and qPCR quantification

RNA was extracted from samples using QIAzol (Qiagen) following the manufacturer’s instructions. cDNA was synthesized using miScript II RT kit (Qiagen). Then, 1μl of a 1:5 diluted cDNA reaction was used in qPCR reactions over 40 cycles using miScript SYBR Green (Qiagen) according to the manufacturer’s protocol. Melt curve analysis was carried out at the end of each run. A list of all primers used in this study are provided in Supplementary Table 1.

### Small RNA-Seq and data analysis

Female mosquitoes (4-day-old) were fed on the calf of a human volunteer for 30 min. Blood-fed mosquitoes with red distended abdomens were identified and transferred into another cage. The fat body of the mosquitoes was dissected from those collected just before blood feeding (NBF; negative control) and 6, 12, and 24 h post blood feeding (hpb). Dissection was done under a stereo microscope by opening the abdomen of the collected mosquitoes in PBS buffer using two pairs of fine forceps as described and visualized previously^51^. Dissected fat body from seven mosquitoes were pooled together as one biological replicate. Small RNAs were extracted from the samples using miRNeasy Mini kit (Qiagen) and treated with Turbo DNase kit (Thermo Fisher) to remove possible genomic DNAs following the manufacturers’ protocols. Two biological replicates were used per treatment and time point. After initial quality control (260/280 ratio and agarose gel analysis), RNA samples were sent to GENEWIZ for Illumina sequencing of small RNAs.

To identify vertebrate blood miRNAs in the fat body tissue sequenced here and also for processing of previously generated mosquito small RNA data^23,24^, the analysis was performed using CLC Genomics Workbench 12.0.3 (Qiagen). The read trimming module was used to process fastq files and low-quality sequences (quality score <0.05), adapter sequences and reads without adapters were removed along with reads <16nt discarded from downstream analysis. High quality reads were then mapped to the *Ae. aegypti* genome (AaegL5.2) to filter out mosquito-related reads using the CLC mapping module under following mapping criteria (mismatch, insertion, and deletion costs: 2:3:3, respectively). Unmapped small RNAs were extracted and counted using small RNA tool from each sample; only miRNAs with a count higher than five were included in the analysis. Human, mouse or chicken miRNAs reported in miRBase v22^52^ were used to annotate the counted small RNAs.

### Protein extraction and purification

For protein extraction, the fat body tissue was macerated in 100 μL of extraction buffer (50 mM Tris-Cl, 150 mM EDTA, 1% (v/v) Igepal (CA-630), 0.1% (v/v) SDS) and then sonicated for 30 sec at 30% power (Qsonica Q125). Subsequently, samples were left on ice for 30 min and then centrifugated at 16,000 g for 10 min at 4 °C. The supernatant was transferred to an Amicon Ultra 0.5 mL filter column (Merck), to which 400 μL of a detergent removal solution (20% methanol-PBS) was added, followed by centrifugation at 14,000 × *g* for 15 min. This step was repeated five times to remove the detergents. Following the last wash, proteins were collected in a Protein LoBind tube (Eppendorf), and the concentration was measured using Direct Detect Infrared Spectrometer (Merck).

Protein samples were then prepared for mass spectrometry using an adapted protocol previously described^53^. Digested peptides with trypsin were separated into two aliquots, half of the sample was used for Sequential Windowed data-independent Acquisition of the Total High-resolution Mass Spectra (SWATH-MS) and the other half for protein identification. For SWATH analysis, 5 μg of the peptides was desalted using 0.6 μL C_18_ ZipTip (Merck) according to the manufacturer’s protocol. Samples were kept at −20 °C until they were ready for mass spectrometry.

For protein identification, 4.5 μg of peptides from each sample was pooled in one tube and separated by high pH reverse phase fractionation using a Sep-Pak C18 1cc Vac Cartridge (Waters) as previously described^54^. Collected fractions were kept at −20 °C until injection.

### Liquid chromatography-mass spectrometry (LC-MS/MS)

Protein samples were separated by reversed-phase chromatography on a Shimadzu Prominence nanoLC system as previously described^55^. Eluted peptides were analyzed on a TripleTOf 5600 instrument (SCIEX) using a Nanospray III interface. MS TOF scan across 350–1800 *m/z* was performed for 0.5 s followed by information-dependent acquisition of up to 20 peptides with intensity greater than 100 counts, across 40–1800 *m/z* (0.05 s per spectra) using collision energy (CE) of 40 ± 15 V. For SWATH analyses, MS scans across 350–1800 *m/z* were performed (0.5 s), and then followed by high sensitivity information-dependent acquisition mode using 26 *m/z* isolation windows for 0.1 sec, across 400-1250 *m/z*. CE values for SWATH samples were automatically assigned by Analyst software (SCIEX) based on *m/z* mass windows.

### Proteome analysis

Peptides and proteins were identified by ProteinPilot v5.0.1 (SCIEX) using the *Ae. aegypti* proteins (AaegL5.2) downloaded from VectorBase^56^. The results from ProteinPilot were used to produce an ion library using PeakView v2.2 (SCIEX). The data output was reformatted with a Python script ReformatMS^57^ and analyzed with MSstats (v2.4)^58^. Protein abundances were compared between Diet-only/miR-21 and NC/miR-21, and only proteins that changed in both comparisons were analyzed further.

### Gene ontology (GO) analysis

GO terms were downloaded for the entire AaegL5 genome from the BioMart server using Gene Stable as ID. Differentially abundant proteins were classified based on their functions using GO terms, over represented GO terms were determined using Blast2GO^59^. An enrichment analysis using Fisher’s Exact Test was done using AaegL5 genome as the universe and the differentially abundant proteins as a test set. Overrepresented terms were considered if they had an FDR lower than 0.05. The GO terms were reduced to a specific term using an FDR filter value of 0.05. A bar chart was produced showing the 10 most significant overrepresented GO terms.

### Target validation using the *GFP* reporter gene

In order to study direct interaction of hsa-miR-21 with *vitellogenin*, a 300 bp product containing two predicted target sites of *vitellogenin A1* (280-580; XM_001657456.2) was amplified. The PCR amplicon was then XbaI and NotI digested followed by its insertion into the similarly digested pSLFa-PUb-MCS plasmid vector (Addgene), which contained the GFP coding sequence upstream of the PCR amplicon insertion site under the *Ae. aegypti polyubiquitin* promoter (pSLFa/GFP-vitell) (Fig. 6D). For confirmation, plasmids were sequenced. A transfection experiment including three biological replicates in Aag2 cell line was conducted where pSLFa/GFP-vitell (500 ng/well in a 12-well plate) was co-transfected with miR-21 mimic, miR-21 mutant 1 (mutated seed region), miR-21 mutant 2 (mutated 3′region) and negative control using Cellfectin as described by the manufacturer (Life Technologies). GFP expression was analyzed through RT-qPCR using *GFP*-specific qPCR primers with three biological replicates after 48 h of transfection. If the mimic interacts with the target, change in the transcript levels of *GFP* are expected.

### Statistics and reproducibility

All statistics were carried out in Graphpad Prism v. 9.1.2. All the source data underlying the graphs are presented in Supplementary Data 2. Significance for differences between treatment and control groups was determined using One-Way ANOVA with Tukey’s post-hoc multiple comparisons. Number of repeated experiments is described in the relevant text or figure legend. Repeated experiments produced consistent results and were considered reproducible.

### Reporting summary

Further information on research design is available in the Nature Research Reporting Summary linked to this article.

## Supplementary information

Supplementary Information

Description of Supplementary Files

Supplementary Data 1

Supplementary Data 2

Reporting Summary

## Data Availability

Small RNA sequencing produced for this study has been deposited in the Sequence Read Archive (SRA) in the NCBI database under the accession number PRJNA685230. Mass spectrometry data have been deposited to the ProteomeXchange Consortium (http://proteomecentral.proteomexchange.org) via the PRIDE partner repository [https://pubmed.ncbi.nlm.nih.gov/30395289/] with the dataset identifier PXD023190.
