# Supplementary material for: Human blood microRNA hsa-miR-21-5p induces vitellogenin in the mosquito Aedes aegypti
Source: Commun Biol. 2021 Jul 9;4:856. doi: 10.1038/s42003-021-02385-7 (PMC8270986; doi:10.1038/s42003-021-02385-7)
Supplement: Supplementary file 1 — Supplementary Information [file 42003_2021_2385_MOESM1_ESM.pdf]

Supplementary Information

Supplementary Table 1. Primers used in this study.

Gene/miRNA	Forward	Reverse
Rps17	CACTCCGAGGTCCGTGGTAT	GGACACTTCGGGCACGTAGT
GFP	CCCAAGCTTCGCCACCATGGTGAGCAA	CGGGGTACCCTTGTACAGCTCGTCCATGC
Vitello target	GGTCTAGACCGTGCCTACCTGGTAATCCG	GGGCGGCCGCCGGATTTGTAAGTGTGGTGACC
Vitello qPCR	TGCTCAGCCCATCGTTTTCT	CGAAGTCGAACACTCCCACA
hsa-miR-451a	AAACCGTTACCATTACTGAGTT	
hsa-miR-16-5p	TAGCAGCACGTAAATATTGGCG	
hsa-miR-21-5p	TAGCTTATCAGACTGATGTTGA	
U6	CGCAAGGATGACACGCAAAAT	
