# Supplementary material for: Human blood microRNA hsa-miR-21-5p induces vitellogenin in the mosquito Aedes aegypti
Source: Commun Biol. 2021 Jul 9;4:856. doi: 10.1038/s42003-021-02385-7 (PMC8270986; doi:10.1038/s42003-021-02385-7)
Supplement: Supplementary file 2 — Description of Supplementary Files [file 42003_2021_2385_MOESM2_ESM.pdf]

Description of Additional Supplementary Files

File name: Supplementary Data 1

Description: Proteins identified by LC-MS/MS in the *Aedes aegypti* female fat body 12 hours after blood feeding.

File name: Supplementary Data 2

Description: Source data underlying the graphs.
